# The Physician’s Visiting List, Diary, and Book of Engagements, for 1861

**Published:** 1861-01

**Authors:** 


ART. X.—*The Physician's Visiting List, Diary, and Book of Engagements, for* 1861. Philadelphia: Lindsay & Blakiston.

*The Physician's Diary for* 1861. Buffalo: Breed, Butler & Co.

*The Physician's Hand-Book of Practice for* 1861. By WILLIAM ELMER, M.D. pp. 200. New York: W. A. Townsend & Co.

THE Physician's Visiting List, issued by Messrs. Lindsay & Blakiston, has been so long before the profession that it is needless to say that it is very useful and convenient to the practitioner. The contents of the book are an almanac; table of signs; Hall's ready method; poisons, and their antidotes; table for calculating the periods of utero-gestation; blank leaves for visiting lists; memoranda; addresses of patients and nurses; obstetric and vaccination engagements; and general memoranda.

The scope of the annual of Dr. Elmer is larger than that of the above work, for, in addition to the contents of the former publication, it comprises a classification of diseases; eruptive and other fevers; diseases of the skin and the other organs of the body; medicinal weights and measures; abbreviation of the medicinal properties of remedies; list of remedial agents; list of incompatibles; medicated baths; writing of prescriptions; and a record of practice and treatment.

The Buffalo publication is very much on the same plan as the work issued in this city, although its contents are not so full. Any of these annuals will prove serviceable to the practitioner.

